# Does the presence of siblings affect the results produced by a surveillance system of child mistreatment? Comparisons of several commonly-used statistical methods

**DOI:** 10.1186/s13104-015-1710-y

**Published:** 2015-12-09

**Authors:** Christelle Senterre, Alain Levêque, Brigitte Vanthournout, Michèle Dramaix

**Affiliations:** Research Centre of Epidemiology, Biostatistics and Clinical Research, School of Public Health, Université Libre de Bruxelles, Brussels, Belgium; SOS Enfants ULB, CHU Saint Pierre, Brussels, Belgium

**Keywords:** Surveillance system, Child mistreatment, Siblings, Clusters, Logistic regression, GEE

## Abstract

**Background:**

Over time, the circumstances encountered in case of child mistreatment, can be quite complex and then, can lead to methodological questions for the analysis of the data. Based on data coming from 395 children hospitalized, alone (66.1 %) or in siblings (33.9 %), in a pediatric ward between 2007 and 2012 for mistreatment or because of a severe risk of mistreatment, the aims of this paper were to quantify the degree of similarity between sibling members, to study the differences between children hospitalized alone or with siblings and to compare four statistical methods (logistic regression and GEE, both without and with robust standard error) for the analyses of the associated factors of mistreatment.

**Results:**

Almost all intracluster correlation coefficients were large, meaning that the sibling’s members have a higher degree of similarity between them. The odds ratios were not exactly the same between the two models and the robust standard errors where almost always higher than the model-based standard errors in both logistic and GEE models leading to wider confidence intervals.

**Conclusion:**

Because many of the intra-siblings correlations observed were relatively strong, the failure to take this cluster dependency into account had a substantial effect on the statistical analyses. Methods taking into account the cluster dependency are widely available in statistical software and strongly recommended.

## Background

Child mistreatment and its associated factors are challenging to estimate because of variation in definitions, but also because of the type of mistreatment being studied, of the comprehensiveness and of the quality of official statistics and of surveys [1]. Lack of good data on the extent, on the associated factors and on the consequences of mistreatments could hold back the development of appropriate responses. Without good and valid data it is difficult to develop a proper awareness of child abuse and neglect and expertise in addressing the problem within the health care, legal and social service professions [2–4]. In Belgium, there is no national data collection program but, in the French speaking region, there is a center, called the Center “SOS enfants ULB” (in English it will be called “SOS children”), within the pediatric ward of the Academic Hospital Center named “CHU Saint Pierre”, which, among others, gives comprehensive care to hospitalized maltreated children [5]. Since 2007, this Center has chosen to systemize and to summarize the data, collected during the children’s stays, through a computerized tool, for the monitoring and the study of several factors associated to child mistreatments. Over time, the circumstances encountered can be quite complex and then, can lead to methodological questions for the analysis of the data. These several circumstances observed can be summarized in four groups: In the first case, the children were hospitalized alone (i.e. without siblings) only one time during the investigated period. In this case, the members of this group are independent of each other and therefore there is no problem to use the usual statistical methods for the analysis. The second case concerns the siblings hospitalized one time during the study period. In this situation, the members of this group are not independent of one another for some variables and therefore, the methods used must be adapted for taking into account the potential cluster effect of the siblings. The third situation concerns the children (alone or with siblings) hospitalized more than one time during the studied period; with consequence that information on the next hospital stay are linked to the first stay, leading to consider a longitudinal (clustered) design. Finally, the last situation is the more complex situations. These complicated situations were, for example, a child hospitalized alone the first time and who came back several months later with a member of his sibling. The inverse situation was also met: the first hospitalization was related to a siblings and the re-hospitalization was only related to one of the sibling’s member. Another example was the situation where two members of a same siblings were hospitalized but not at the same time.

Based on these several situations, the main objective of this paper was to study the potential effect of siblings’ presence in the dataset; therefore the three aims pursued were to (1) quantify the degree of similarity among sibship members, (2) study the differences of characteristics between the children hospitalized alone and the children hospitalized with siblings and (3) compare several commonly-used statistical methods which take into account (or not) the within-siblings potential similarities for the analyses of the associated factors to the mistreatment of the children. Note that this paper is designed to present an overview of the application of several commonly-used statistical methods to “real world” data. It is not intended to provide information on the statistical theory behind the models.

## Methods

### Data source and variables used

Between 2007 and 2012, there were 504 hospitalizations of children in the pediatric unit of the CHU; with a yearly number almost steady (n_2007_ = 91, n_2008_ = 73, n_2009_ = 85, n_2010_ = 79, n_2011_ = 90 and n_2012_ = 86). Because this paper is focused on the potential effect of siblings’ presence (and not focused on the repeated hospitalizations), the choice was made to only include the children hospitalized only one time during the studied period. So, the database used in this paper contained the information from the 395 children hospitalized, alone (66.1 %) or in siblings (33.9 %), in the pediatric ward between 2007 and 2012 for mistreatment or because of a severe risk of mistreatment. This study doesn’t require an ethical approval because the data is collected under the responsibility of one of the co-authors and analyzed directly by the principal author to produce a part of the annual report of the Center “SOS enfants ULB”.

Upon arrival and throughout the stay, a series of information are collected about the children and their situations [5]. This information concerns some socio-demographic characteristics (age, gender, living environment before the hospitalization). If the child was hospitalized with siblings it was marked (note that each member of the sibship has his own record in the dataset resulting from the encoding in the computerized tool). Some information about known previous case files were also reported. These previous files could be (or not) files opened previously by the SOS enfants ULB team or before the Court, through a Juvenile Judge or a Youth Service (but, as a reminder, in this paper, there are no child who had a record of a previous hospitalization). Due to the social vocation of the hospital and the fact that it is located in Brussels, the team encounters sometimes language barrier with the children and their relatives. So, the intervention of a translator was also reported. Concerning the factors taken on a share of hospitalizations, four types were reported: they concern medical and psychiatric aspects of the children but also parental factors and especially the potential protective factors implicated. During the stay, the mistreatment checkup is done and a personality diagnosis is established. Finally, at the discharge, the return (or not) in the living environment and the possible measures taken were also reported. All the collected information was not used in this paper, so we present the retained variables (main outcomes investigated and associated factors) just below.

The main outcome was a dichotomous variable coded as (1) for the children who were diagnosed as mistreated and (0) for the children who were diagnosed as at risk (of mistreatment). The six associated factors investigated were the gender; the age group (≤3 vs. ≥4 years); the country of birth (with children born in Belgium vs. those born in another country); the living environment before the hospitalization (children living in family vs. those who live elsewhere); the type of hospital admission (admissions through the emergency department vs. planned admissions) and, finally, the existence of known previous case files (have one or more known previous case files vs. not having). These files can correspond to mistreatment history known by a Health Youth Service or by Juvenile Judge and/or Court [5].

### Statistical analyses

To quantify the degree of similarity between sibship members, the Snijders and Bosker intracluster correlation coefficient (ICC) was calculated for the binary main outcome (mistreatment or at risk of mistreatment) and for each of the associated factors (gender, age, country of birth, living environment, type of admission and known previous case files). To obtain this ICC, two-level unconditional (variance-components) logistic models were undertaken, only for children hospitalized in siblings, with members of the siblings as level 1 and siblings as level 2. In this type of model, the total variance is the sum of the variance between clusters, i.e. the siblings, and the variance within clusters, i.e. between the members of the same sibling [6, 7]. Values of the ICC range from 0 to 1. When the within-cluster variance moves towards 0, meaning that all the subjects of the cluster (i.e. the members of the siblings) are similar, the ICC gets closer to 1; and when the within-cluster variance is much greater than the between-cluster variance the ICC gets closer to 0 [8]. The likelihood ratio (LR) test, which compares the likelihood from the logit model to the likelihood from two-level variance-components model, was used to obtained the degree of statistical significance of the ICC’s [7].

In the results section, the intracluster correlation coefficients, with their 95 % confidence intervals, and the p value of the LR test were reported in a Table 1.

**Table 1:** Values of the intracluster correlation coefficients among the siblings for the mistreatment status and for the six associated factors investigated (n = 134, with 57 siblings and with minimum 2 and maximum 6 children by siblings)

Variables	ICC (95 % CI)	P value_LR_
Status (mistreatment vs. at risk)	0.888 (0.694–0.964)	<0.001
Gender	≈0	1
Age	0.272 (0.064–0.670)	0.042
Country of birth	0.873 (0.730–0.946)	<0.001
Living environment	0.857 (0.724–0.932)	<0.001
Type of admission	0.986 (0.968–0.994)	<0.001
Previous known case files	0.996 (0.994–0.997)	<0.001

The P value_LR_ is the p value from the likelihood ratio test

To study the differences of characteristics between the children hospitalized alone and the children hospitalized with siblings, the comparisons of the proportions of the main outcome and of the six associated factors, were made, on one hand, with the help of the Pearson’s Chi square test and on the other hand, to take into account the presence of clustered data, with the help of the Pearson’s Chi square test with the second-order correction of Rao and Scott [9, 10].

In the results section, Table 2 presents, on one hand the proportions of the main outcome and of the six associated factors and on the other hand the p values of the two undertaken Chi square tests.

**Table 2:** Characteristics of the children according to the type of hospitalization (children hospitalized alone or with siblings)

Variables	Total (n = 395)	Hospitalized alone (n = 261)	Hospitalized in siblings (n = 134)	P value_P_	P value_PRS_
Status				0.262	0.404
Mistreatment	46.1	44.1	50.0		
At risk of mistreatment	53.9	55.9	50.0		
Gender				0.700	0.696
Girls	50.9	50.2	52.2		
Boys	49.1	49.8	47.8		
Age				0.114	0.144
≤3 years	46.6	49.4	41.0		
≥4 years	53.4	50.6	59.0		
Country of birth				0.243	0.397
Belgium	78.7	77.0	82.1		
Other country	21.3	23.0	17.9		
Living environment				0.026	0.111
Elsewhere	9.9	12.3	5.2		
Family	90.1	87.7	94.8		
Type of admission				0.192	0.334
Planned	18.5	20.3	14.9		
Emergency	81.5	76.7	85.1		
Previous known case files				<0.001	0.001
No	54.7	62.8	38.8		
Yes	45.3	37.2	61.2		

The P value_P_ is the p value from the Pearson’s Chi square test

The P value_PRS_ is the p value of the Pearson’s Chi square test with the Rao and Scott correction

To analyze the possible effect of siblings’ presence on the associations between the main outcome and the potential associated factors, four marginal models were tested: the standard logistic regression, the logistic regression with clustered robust standard error, the generalized estimating equation (GEE) and the GEE with robust standard errors. These four models are population-averaged approaches [11].

*[M1] The standard logistic regression* With a binary outcome, usually coded 0/1, where 1 can be interpreted as the occurrence of the event and 0 as the non-occurrence of event, the logistic regression model provides a simple and plausible way to estimate the probability of occurrence of the event. By derivation, it is possible to demonstrate that the exponential of the regression coefficient corresponds to the odds ratio, a commonly used measure to estimate the strength of the associations [12]. In this paper, the odds ratio will be then, the odds of mistreatment (vs. at risk of mistreatment) among the “exposed” group divided by the odds of it among the “reference” group. Theoretically, the standard logistic regression model assumes that all the observations are independent [13]. Violations of the assumption of independence of observations may result in incorrect statistical inference due to biased standard errors [14, 15].

*[M2] The logistic regression with clustered robust standard error* This model is the same as [M1] but the traditional standard errors are replaced by robust standard errors, which are also known as Huber and White (sandwich) standard errors. These robust estimators allow for take account of the intracluster correlation, relaxing the assumption of independence of the observations. In this model, only the standard errors and the *p* value are affected and the estimated coefficients are the same than those obtained by simple logistic regression [13].

*[M3] The generalized estimating equation (GEE)* Liang and Zeger [16] have proposed the generalized estimating equations as an extension of the generalized linear model to take into account the correlation between observations. A GEE model requires to specify (a) the link function to be used, (b) the distribution of the dependent variable, and (c) the “working” correlation structure of the dependent variable among the subjects of the clusters [11, 17–19]. When logit function is chosen as the link function and when the binomial family is chosen to characterize the distribution of the dependent variable, the model corresponds to a logistic regression. Consequently, the exponential of the regression coefficient corresponds also to the odds ratio [11, 18, 20]. Nevertheless, because the computational algorithm of the estimators is different; estimated regression coefficients will typically be different from those obtained with a logistic regression model [7]. Regarding the choice of the correlation structure, an exchangeable correlation structure is often more appropriate for clustering at family level [17–19, 21] This exchangeable correlation structure assumed the same correlation for all pairs of subjects, reflecting average dependence among the observations in the same cluster. In our case, this should mean that the relation between the members of the same siblings is assumed to be equally correlated [11].

*[M4] The GEE with robust standard errors* The GEE models, which use the model-based variance estimation, could lead to biased estimations of the standard errors in case of misspecification of the correlation structure. Therefore, the use of GEE models with robust standard errors, using the Huber and White (sandwich) estimator of variance, leads to produce valid standard errors even in the event of misspecification of the correlation structure [7, 14, 16, 17, 19].

In the results section, Table 3 presents, for the four modeling approaches: the odds ratio of the mistreatment among the six investigated factors, derived from the models, with their standard errors, their 95 % confidence intervals and the p value of the Wald’s tests. Finally, for taking into account the potential confounding effects of the investigated factors among them, the four models were started again under a multivariable approach; therefore, Table 4 presents the adjusted odds ratios, also with their standard errors, their 95 % confidence intervals and the p-value of the Wald’s tests.

**Table 3:** Bivariate analysis of the associated factors according to the mistreatment status

Variables (n = 395)	Logistic	Logistic robust	GEE	GEE robust
	OR (ES) (95 % CI)	OR (ES) (95 % CI)	OR (ES) (95 % CI)	OR (ES) (95 % CI)
Gender	*P* = *0.494*	*P* = *0.502*	*P* = *0.809*	*P* = *0.838*
Girls	1.00	1.00	1.00	1.00
Boys	1.15 (0.23) (0.77–1.71)	1.15 (0.24) (0.77–1.72)	0.96 (0.15) (0.70–1.31)	0.96 (0.18) (0.67–1.39)
			[Correlation matrix: 0.770]	
Age	*P* < *0.001*	*P* < *0.001*	*P* < *0.001*	*P* < *0.001*
≤3 years	1.00	1.00	1.00	1.00
≥4 years	3.42 (0.72) (2.26–5.18)	3.42 (0.76) (2.22–5.29)	2.40 (0.38) (1.76–3.29)	2.40 (0.53) (1.56–3.72)
			[Correlation matrix: 0.839]	
Country of birth	*P* < *0.001*	*P* = *0.001*	*P* = *0.001*	*P* = *0.001*
Belgium	1.00	1.00	1.00	1.00
Other country	2.58 (0.69) (1.53–4.36)	2.58 (0.76) (1.45–4.59)	2.28 (0.59) (1.38–3.77)	2.28 (0.59) (1.38–3.78)
			[Correlation matrix: 0.730]	
Living environment	*P* = *0.001*	*P* = *0.006*	*P* < *0.001*	*P* < *0.001*
Elsewhere	1.00	1.00	1.00	1.00
Family	3.34 (1.24) (1.61–6.92)	3.34 (1.48) (1.40–7.95)	4.72 (1.80) (2.23–9.99)	4.72 (2.21) (1.89–11.79)
			[Correlation matrix: 0.843]	
Type of admission	*P* = *0.004*	*P* = *0.006*	*P* = *0.006*	*P* = *0.003*
Planned	1.00	1.00	1.00	1.00
Emergency	2.17 (0.58) (1.29–3.65)	2.17 (0.61) (1.25–3.77)	2.12 (0.58) (1.24–3.62)	2.12 (0.54) (1.29–3.49)
			[Correlation matrix: 0.768]	
Previous known case files	*P* = *0.757*	*P* = *0.795*	*P* = *0.667*	*P* = *0.686*
No	1.00	1.00	1.00	1.00
Yes	0.94 (0.19) (0.63–1.40)	0.94 (0.23) (0.59–1.51)	0.91 (0.20) (0.60–1.39)	0.91 (0.21) (0.58–1.43)
			[Correlation matrix: 0.777]	

All values are vs. at risk of mistreatment. The “P” are the p values of the Wald tests

The “Correlation matrix” is the estimated (exchangeable) within-sibship correlation matrix

**Table 4:** Multivariable analysis of the associated factors according to the mistreatment status

Variables (n = 395)	Logistic	Logistic robust	GEE	GEE robust
	OR_a_ (ES) (95 % CI)	OR_a_ (ES) (95 % CI)	OR_a_ (ES) (95 % CI)	OR_a_ (ES) (95 % CI)
			[Correlation matrix: 0.835]	
Gender	*P* = *0.342*	*P* = *0.935*	*P* = *0.956*	*P* = *0.968*
Girls	1.00	1.00	1.00	1.00
Boys	1.23 (0.27) (0.80–1.88)	1.23 (0.27) (0.80–1.89)	1.01 (0.15) (0.75–1.36)	1.01 (0.21) (0.67–1.52)
Age	*P* < *0.001*	*P* < *0.001*	*P* < *0.001*	*P* = *0.002*
≤3 years	1.00	1.00	1.00	1.00
≥4 years	2.77 (0.63) (1.77–4.32)	2.77 (0.69) (1.70–4.50)	2.06 (0.36) (1.47–2.89)	2.06 (0.49) (1.29–3.28)
Country of birth	*P* = *0.052*	*P* = *0.088*	*P* = *0.115*	*P* = *0.139*
Belgium	1.00	1.00	1.00	1.00
Other country	1.76 (0.51) (1.00–3.10)	1.76 (0.58) (0.92–3.36)	1.50 (0.39) (0.91–2.50)	1.50 (0.42) (0.88–2.59)
Living environment	*P* = *0.020*	*P* = *0.031*	*P* = *0.002*	*P* = *0.014*
Elsewhere	1.00	1.00	1.00	1.00
Family	2.57 (1.04) (1.16–5.68)	2.57 (1.12) (1.09–6.04)	3.57 (1.45) (1.60–7.93)	3.57 (1.85) (1.29–9.85)
Type of admission	*P* = *0.260*	*P* = *0.294*	*P* = *0.323*	*P* = *0.309*
Planned	1.00	1.00	1.00	1.00
Emergency	1.39 (0.41) (0.78–2.48)	1.39 (0.44) (0.75–2.59)	1.34 (0.40) (0.75–2.40)	1.34 (0.39) (0.76–2.36)
Previous known case files	*P* = *0.900*	*P* = *0.919*	*P* = *0.831*	*P* = *0.844*
No	1.00	1.00	1.00	1.00
Yes	0.97 (0.21) (0.63–1.50)	0.97 (0.26) (0.57–1.66)	1.05 (0.24) (0.67–1.64)	1.05 (0.26) (0.65–1.71)

All values are vs. at risk of mistreatment. OR_a_ are the adjusted odds ratio. The “P” are the p values of the Wald tests

The “Correlation matrix” is the estimated (exchangeable) within-sibship correlation matrix

The significance level for all tests was 0.05 and all statistical analyses were performed using Stata/SE 12.0 for Windows (TX: StataCorp LP).

## Results and discussion

### Description of the several siblings and degree of similarity between the sibling members

The 134 children hospitalized in siblings were allocated in 57 siblings with a minimum of children by sibling equal to 2 and a maximum equal to 6. There were 44 siblings of 2 children (with 5 pairs of twins among these); 8 siblings of 3 children; 4 siblings of 4 children and 1 sibling of 6 children. In other words, the clusters’ size in this study is relatively small and it is known that the ICC tends to be larger for smaller clusters [22]. On the other hand, the number of siblings i.e. the clusters tends to be sufficiently large for GEE methods to be applicable [20]. Table 1 shown that all the ICC’s, except those ones for the gender and for the age, were nearly equal to 1, meaning that the members of the siblings have a higher degree of similarity between them. The thorough descriptive study of the data shown that among the 57 siblings, 10 were heterogeneous for the status of mistreatment: in 8 siblings of 2, one of children was been diagnosed as a mistreated child and the other one as a child at risk of mistreatment. In a sibling of 3 children, 2 were mistreated and one was considered at risk of mistreatment; and finally, in a sibling of 4 children, 1 was mistreated and the 3 others were considered as at risk of mistreatment. Regarding the country of birth, discrepancies within siblings were observed 7 times on 57; with at each time, one of two children born in Belgium for the 3 siblings of 2 children, one of three children born in Belgium for the 3 siblings of 3 children and 1 child born in Belgium in the siblings of 6 children. Finally, regarding the living environment, only two discrepancies were observed (for two siblings of two children), at each time, one of the two children was reported as not living in the family. These two situations corresponded to children who were born at the hospital and who were directly transferred in the pediatric ward because there were considered as at risk of mistreatment (with the other child of the siblings diagnosed as mistreated).

### Differences of characteristics between the children hospitalized alone or with siblings

Table 2 shown that, regarding the status of mistreatment, the proportion of children at risk of mistreatment is a little more higher in the group of children hospitalized alone but the difference was not statically significant both with result of the Pearson’s Chi square test uncorrected and the corrected test for take into account the presence of clustered data. About the potential associated factors to mistreatment, only the previous known case files (with more previous case files for siblings) were statistically significantly different between children hospitalized alone or in siblings both with the two Chi square tests (uncorrected and corrected). In these two situations, because the p-values were unhesitatingly either statistically significant or not, the use of the Rao and Scott correction have not changed the interpretation of the results. On the other side, for the living environment, not take into account the correction for clustered data lead to a wrong conclusion.

### Comparisons of the several statistical methods

The results presented in the Table 1 have shown that the siblings were relative homogenous and the results presented in the Table 2 have not shown major differences between the children hospitalized alone or in siblings regarding the main outcome and in terms of associated factors, excepted for the previous known case files. In some studies, authors have made the choice to only keep the data from one child by sibling enrolled; another have made the choice to reduce the data to a single measurement per cluster [23, 24]. These two approaches reduce both the amount of information and the number of subjects included in the analyses. Consequently, it seems to be better to analyze the relations between the main outcome and the potential associated factors without reducing the number of children and without separating the group of hospitalized alone from those hospitalized with siblings. Tables 3 and 4 present the results of the four modeling approaches undertaken.

As stated in the methodology, because the computational algorithm of the estimators is different between the logistic models and the GEE models, the odds ratio estimates were not exactly the same between the two groups of models. Considering the standard errors from both the logistic models and the GEE models, those from the robust models were higher than those observed in the basic forms of the models; leading to larger confidence intervals (excepted for the country of birth and the type of admission in the univariate GEE models; the gender in the multivariable logistic models and the type of admission in the multivariable GEE models). Regarding the investigated factors, the age, the country of birth, the living environment and the type of admission were statistically significantly associated, in the univariate analyses, to the status of mistreatment, independently of model undertaken. In the four models, the p values of the Wald tests were unhesitatingly significant. In the multivariable analyses, only age and living environment stayed statistically significantly associated to the status of mistreatment.

## Conclusion

The three aims pursued were to quantify the degree of similarity between the members of the sibships, to study the differences of characteristics between the children hospitalized alone and the children hospitalized with siblings and to compare four commonly-used statistical methods which take into account (or not) the within-siblings potential similarities for the analysis of the associated factors to the mistreatment of the children. Three of the four modeling approaches adjust for clustering in some way: firstly, the logistic-robust approach (which is equivalent to GEE with a working independence correlation matrix with robust standard errors) takes the clustering into account via use of the sandwich variance formula; secondly, the GEE with exchangeable correlation and model-based variance estimator takes clustering into account with the exchangeable correlation, however this correlation model must be correctly specified for inference with the model-based standard errors to be valid; and thirdly, the GEE with exchangeable correlation and robust standard errors takes clustering into account both ways (in the computational algorithm producing the odds ratio estimates and in the variance estimation with the sandwich estimator).

It is known that the effect of the clusters depends of the strength of the intracluster correlations [23].

Because some of the intra-siblings correlations observed were relatively strong, the failure to take this cluster dependency into account has had a potential effect on the statistical analyses (through incorrect estimations of the standard errors, of the confidence intervals and of the inference). Therefore, it is clear that the standard logistic regression model is not appropriate; and among the three other modeling approaches (which take into account the presence of clusters), the GEE with robust standard errors seems the most appropriate to protect against misspecification of the exchangeable correlation assumption.

Finally, because of a large number of the statistical software proposes robust models, using methods taking into account the cluster dependency is feasible and useful.
